# Pediatric lipomas: Usual sights-unusual sites

**DOI:** 10.1016/j.ijscr.2025.111822

**Published:** 2025-08-19

**Authors:** Ashish Lal Shrestha, Devendra Bist, Gandham Edmond Jonathan

**Affiliations:** a Department of Pediatric Surgery, Kathmandu Medical College and Teaching Hospital, Kathmandu, Nepal; b Department of Neurosurgery, Nobel Medical College and Teaching Hospital, Biratnagar, Nepal

**Keywords:** Pediatric lipomas, Surgical excision, Rare sites, Benign tumors, MRI diagnosis

## Abstract

Pediatric lipomas are uncommon, comprising less than 10 % of all soft tissue tumors in children. While they commonly occur in subcutaneous tissues, their presence in atypical locations can pose diagnostic challenges.

We present a series of three children with lipomas at unusual anatomical locations namely: ankle, supraclavicular and dorsolumbar regions. Each case was evaluated clinico-radiologically, followed by complete surgical excision and histopathological confirmation. All had uneventful postoperative recovery and remained symptom free at 3 years follow up.

## Highlights

• Pediatric lipomas are rare, representing <10 % of pediatric soft tissue tumors.
• Unusual anatomical sites may mimic malignancy and require imaging.
• Complete surgical excision is curative and confirms diagnosis.
• Three rare site lipomas presented: ankle, supraclavicular, dorsolumbar.
• All patients had excellent outcomes with no recurrence at 3 years.

## 1 Introduction

Lipomas, the benign tumors composed of mature adipose tissue are amongst the most common soft-tissue neoplasms encountered in surgical practice. In adults, they predominantly arise in the subcutaneous tissues of the trunk and extremities and seem to follow a predictable clinical course [1]. However, in children and upto the first two decades of life, lipomas account for fewer than 10 % of all the soft tissue tumors [2]. Pediatric lipomas are uncommon compared to vascular tumors, and liposarcoma is rarely reported in young children [3].The trunk is the commonest site wherein the subcutaneous fat is the commonest location although a few may be encountered in the deeper tissues such as the intramuscular, intermuscular or submuscular planes [4].

Complete surgical excision remains the mainstay of treatment for symptomatic lipomas, effectively removing them and thereby confirming the diagnosis through histopathology which also aids in ruling out malignancy or other conditions [5,6].

This case series aims to document the clinical and surgical experience with pediatric lipomas presenting in atypical anatomical sites, focusing on their management strategies and outcomes.

## 2 Case description

### 2.1 Case 1: Posterior ankle lipoma

**Fig. 1:**
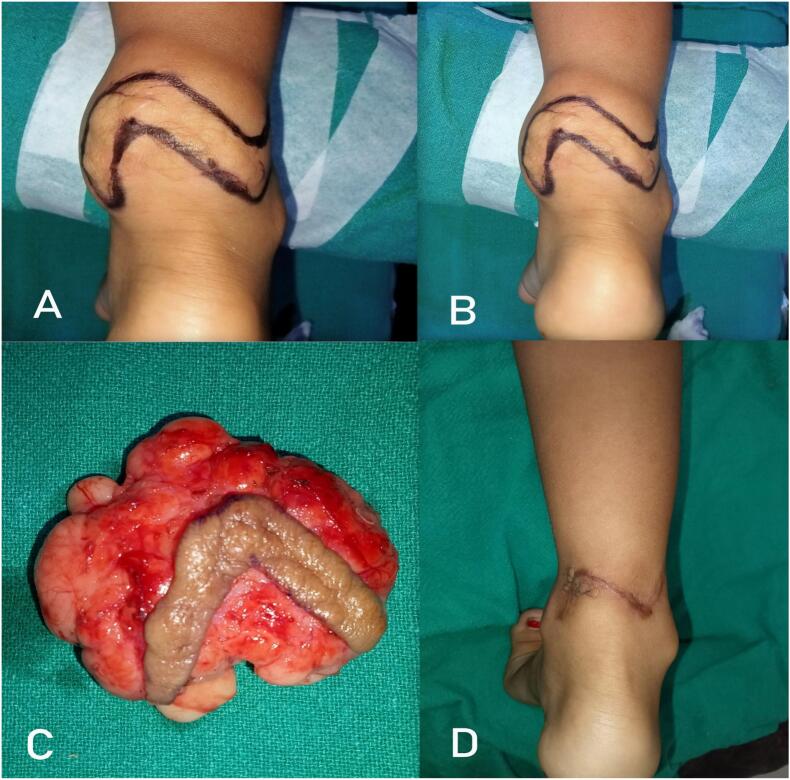
Recurrent posterior ankle lipoma A and B: Clinical Picture, C: Excised specimen, D: Follow up at 6 months.

A 6 year-old girl presented with a recurrent painless swelling over the posterior aspect of right ankle, progressively increasing in size over past several months. She had undergone an earlier excision elsewhere a year ago. Clinical examination revealed a soft, non tender, non compressible, well-circumscribed lump with a lobulated surface measuring approximately 6 × 5 cm over the posterior aspect of the right ankle joint in the sub cutaneous plane with a linear scar of previous operation on the overlying skin. Ultrasonography(USG) favored a clearly subcutaneous lipomatous lesion without articular extension. Therefore, without further imaging, complete surgical excision was planned and performed under general anesthesia along with the scar overlying the lesion as shown in Fig. 1. Intra operative appearance was that of a lobulated fatty encapsulated tumor resembling a lipoma.

**Fig. 2:**
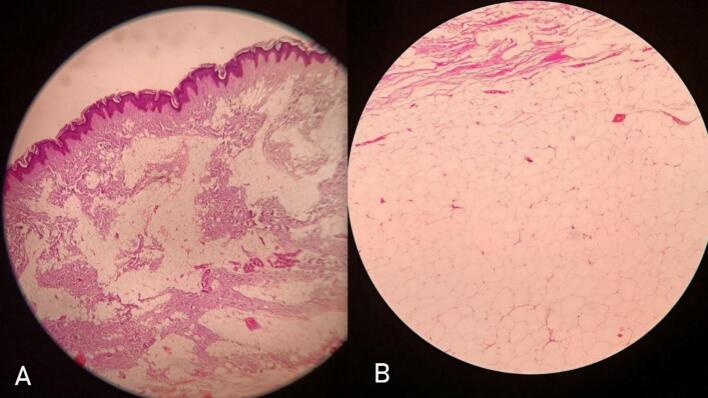
A. Histopathological examination of the recurrent posterior ankle lipoma stained with Eosin and Haematoxylin A: 4× Magnification B: 10× Magnification.

Histopathologically, gross examination confirmed the operative findings and the cut section revealed a solid, firm and greasy material. Microscopically, the encapsulated mass comprised of lobules of adipocytes separated by fibrous septae. The adipocytes had abundant vacuolated cytoplasm with eccentrically placed flattened nuclei. The findings confirmed a lipoma as shown in Fig. 2. She had an uneventful postoperative recovery. At three years follow up she remained asymptomatic and recurrence free.

### 2.2 Case 2: Supraclavicular lipoma

**Fig. 3:**
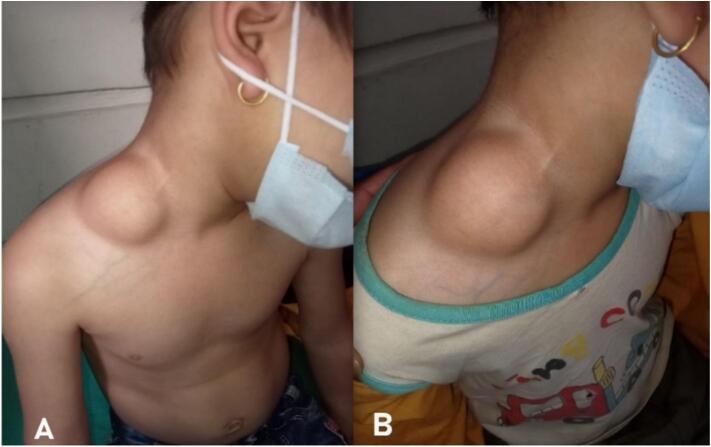
A. and B: Clinical picture of right supra clavicular lipoma.

A 4 year old boy presented with a painless slowly progressive swelling over the right side of the neck noticed 20 days prior to evaluation. Clinical examination revealed a single, soft, nontender, non compressible lump with distinct margins and lobulated surface in the sub cutaneous plane of the posterior triangle of neck over the right side measuring approximately 4 × 4 cm with an intact overlying skin as shown in Fig. 3. The USG revealed a well-defined cervical lesion measuring 4X4X2 cm in the subcutaneous plane.

**Fig. 4:**
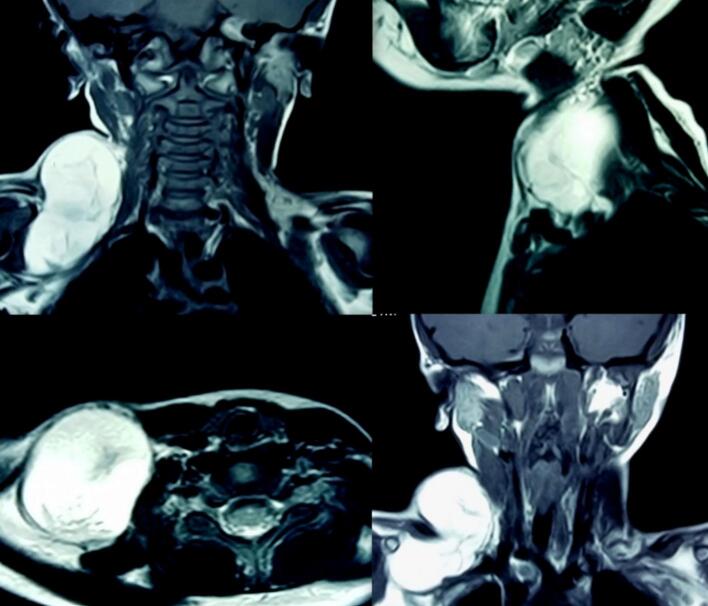
Plain MRI Findings of right supraclavicular lipoma.

Plain Magnetic Resonance Imaging (MRI) neck done to assess the deeper extent and structural relations of the lesion showed an “**8”** shaped fat signal intensity in the supraclavicular region measuring 7X3X4cm with an infraclavicular extension. The lesion was peripherally capsulated and compressing the elements of brachial plexus and subclavian vessels inferiorly, abutting sternomastoid muscle anteromedially, scalene muscles medially and trapezius muscle posteriorly. The features were consistent with a lipoma as shown in Fig. 4.

**Fig. 5:**
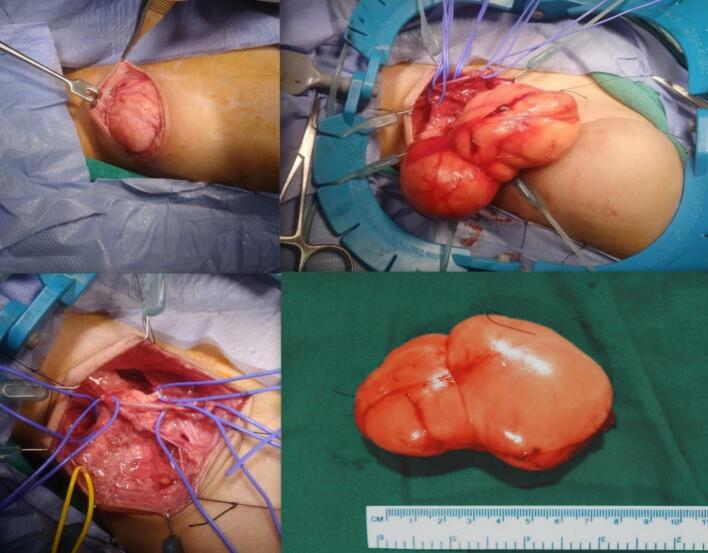
Intra operative findings of right supra clavicular lipoma.

He underwent complete surgical excision of the lesion under general anesthesia, with preservation of jugular veins, subclavian vessels, brachial plexus and accessory spinal nerve. The intra operative appearance was that of a lipomatous lesion as shown in Fig. 5. Postoperative period was uneventful and follow-up evaluation was unremarkable. The histopathology confirmed a lipoma. At three years follow up, he remained asymptomatic and recurrence free.

### 2.3 Case 3: Dorsolumbar lipoma

**Fig. 6:**
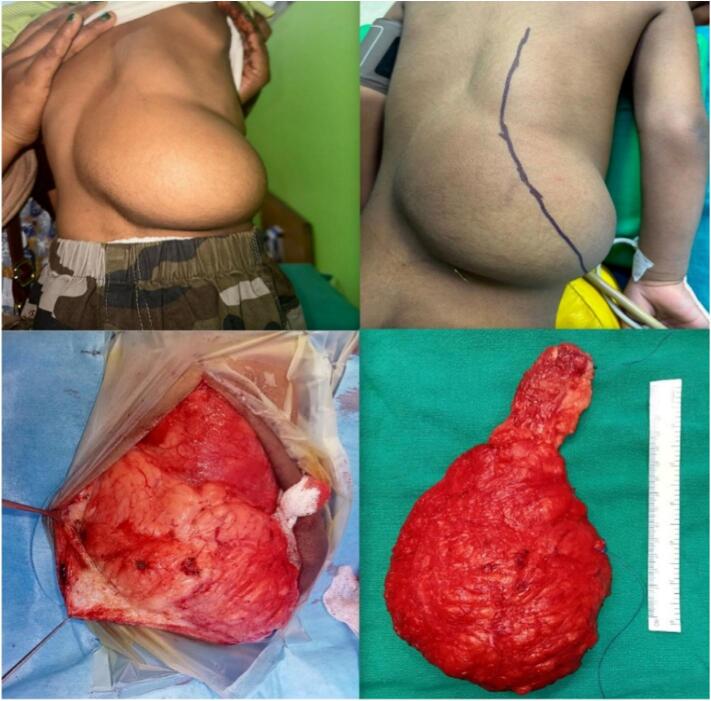
Clinical and Intraoperative findings of right dorso lumbar lipoma.

**Fig. 7:**
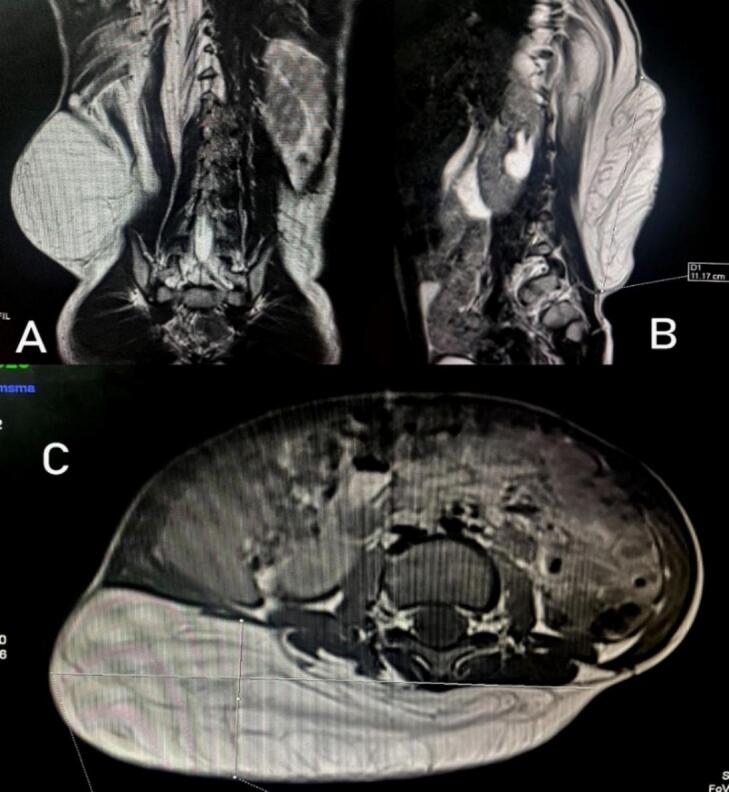
MRI findings of right dorso lumbar lipoma.

A 2 year old boy was evaluated for a painless slowly progressive swelling over the back in the right dorsolumbar region for a year. Examination revealed a large, soft, non-tender, non compressible mass with variegated surface and poorly defined borders as shown in Fig. 6. The mass was approximately 30 × 10 cm in the sub-cutaneous plane over most of its extent except for the superior portion that seemed to be merging with the underlying muscles. There were no neurological deficits. The lesion was suspected to be a liposarcoma, given its size and deep-seated nature. MRI showed diffuse infiltration of fat signal within the right paraspinal muscle in dorsolumbar region extending from D7-L4 disc level interdigitating with right paraspinal muscle fibers causing its enlargement and extending into the D10-L1 neural foramina as shown in Fig. 7.The features were consistent with a lipoma with infiltration into the deeper muscles over the superior aspect. Complete surgical excision under general anesthesia was performed, revealing a large lipomatous lesion measuring approximately 30 × 10 cm, weighing 600 g, with infiltration into the superior two-thirds of the right paraspinal muscles although staying clearly off the spinal canal or neural foramina. His postoperative period was uneventful and follow-up evaluation was unremarkable. The lesion was confirmed to be lipoma on histopathology. At 3 years follow up, he continued to remain asymptomatic and recurrence free.

## 3 Discussion

Lipomas hitherto considered the commonest of the adult soft tissue tumors can practically exist at all the sites where fat can occur, earning them the title of “Ubiquitous Tumors” [7]. However, in children, they are considered to be relatively rare making up for up to less than 10 % of soft tissue tumors and often preceeded by tumors of vascular origin [8].This is consistent with findings from a large study in children, noting lipomas to comprise 15.6 % of benign soft tissue tumors while 48.6 % being hemangiomas [9].

Composed entirely of mature adipocytes, the usual sites for lipomas are the trunk and the proximal extremities where they are often located in the subcutaneous plane, although their occurences in variable depths are also known. When encountered at atypical anatomical locations (particularly in children) or at “**unusual sites”** such as colon; presenting with obstruction, thereby masquerading malignancy they can pose significant diagnostic challenges [7]. The current series highlights the importance of considering lipomas based on clinico-radiological grounds in order to avoid misdiagnosis and possibilities of a “Whoops' Procedure” [10].

The usual findings of a slow growing well defined lump with a lobulated surface in the subcutaneous plane at a “**usual site**” is a “**usual sight**” but nevertheless, a detailed imaging like an MRI may be required to rule out more sinister lesion like Lipoblastoma, Liposarcoma or even a deeply seated intramuscular or infiltrative variant based on clinical indication.

Lipoblastoma a pediatric specific adipocytic tumor often mimics lipoma on imaging but contains immature adipocytes and myxoid matrix, requiring histopathological confirmation [11]. Likewise, distinction from liposarcoma is critical, though the latter is exceedingly rare in children under 10 years [3]. A study on pediatric soft tissue tumors worked up with a suspicion of sarcoma eventually confirmed 59.5 % to be non-neoplastic, the commonest being a vascular malformation, emphasizing the need for rigorous imaging to avoid over-treatment [12]. On the other hand, variants like Angiolipoma and Neurolipoma are also well described.

Additionally, Juxta articular lipomas at the ankle are exceedingly rare with limited case reports involving only the adult population [13]. With Case 1, although recurrence was a concern, in view of clinical findings, complete surgical excision was attempted and the histopathological confirmation reenforced the supposition that the lesion was probably only partially excised at first procedure causing a subsequent recurrence.

Similarly, supraclavicular location for lipomas are again rare in the pediatric population and even rarer with an infraclavicular extension. A similar report of a 10-month-old was noted with an asymptomatic neck mass wherein complete surgical excision was achieved without complications [14]. With Case 2, based on clinico-radiological information, a complete surgical extirpation could be carried out at the same time preserving vital structures in the viscinity in the neck, the histopathology reconfirming a lipoma. Finally, dorsolumbar lipomas require careful assessment due to their potential associations with spinal anomalies [15]. A study analyzing 50 pediatric cases of lipomatous tumors with 7.7 % over the spinal and lumbosacral region, found that lipomas accounted for 52 % of cases, necessitating thorough evaluation to distinguish them from malignant counterparts [16]. With Case 3, a giant (>10 cm) dorsolumbar lipoma, MRI was useful to confirm muscular infiltration at its superior border and to rule out intraspinous extension resulting in a successful in toto excision. The histopathological examination once again confirming a lipoma.

Regardless of site, a slow growing tumor even with features resembling a lipoma can still be a concern in view of pressure effects that is imposed upon the surrounding structures of vital importance. A sudden rapid proliferation in a relatively slow growing lipomatous lesion could raise concerns regarding a malignant transformation. These are the usual indications for intervention on a supposedly lipomatous lesion if not for pain or cosmesis.

Complete surgical excision with intact capsule is the mainstay of definitive treatment providing both relief and histopathological confirmation. With above series, we share our experiences that align with global reports, indicating excellent prognoses post-excision with minimal recurrence rates [17].

This case series has been reported in line with the PROCESS Guidelines [18].

## 4 Conclusion

Pediatric lipomas, though benign can pose diagnostic and therapeutic challenges when encountered at atypical locations. Our case series underscores the necessity of comprehensive clinical and imaging evaluations to accurately diagnose and manage these lesions.

Complete surgical excision offers definitive treatment with excellent outcomes. Further studies and long-term follow-ups are essential to deepen the understanding of their behavior at various anatomical sites.

## Consent

Written informed consent was obtained from the patients' guardians for publication of this case series and accompanying images. A copy of the written consent is available for review by the Editor-in-Chief of this journal on request.

## Ethical approval

Ethical approval was not required for this case series as per the policies of the Kathmandu Medical College and Teaching Hospital, since the work did not constitute research but rather clinical case documentation and analysis. The study involved retrospective analysis of anonymized patient data and written informed consent was obtained from the guardians for publication.

## Funding

No funding was received for this study.

## Author contribution

Ashish Lal Shrestha: Concept, manuscript writing, surgical management. Devendra Bist: Data collection, literature review, original draft writing. Gandham Edmond Jonathan: Manuscript review, neurosurgical evaluation, surgical management.

## Guarantor

Dr. Ashish Lal Shrestha.

## Research registration number

Not applicable.

## Conflict of interest statement

There are no conflicts of interest.
